# A New Determination Method of the Solubility Parameter of Polymer Based on AIE

**DOI:** 10.3390/molecules22010054

**Published:** 2016-12-30

**Authors:** Shan Jiang, Tian Ya Huang, Ke Min Wang, Ben Zhong Tang, Qiang Yu

**Affiliations:** 1 School of Materials Science and Engineering, Changzhou University, Changzhou 213164, China; Jiangshan@cczu.edu.cn (S.J.); 14101302@smail.cczu.edu.cn (T.Y.H.); wangkm61@gmail.com (K.M.W.); 2 Department of Chemistry, The Hong Kong University of Science & Technology, Clear Water Bay, Kowloon, Hong Kong, China; tangbenz@ust.hk

**Keywords:** aggregation-induced emission, conformation transition, luminescence, solubility parameter

## Abstract

An accurate method of the fluorescence probe approach based on an aggregation-induced emission (AIE) molecule (tetraphenylethylene) for measuring the solubility parameter of the polymer is reported. This method is distinctive in that the approach can make the polymer chain conformation in solution be related to the fluorescence intensity. Since the solubility parameter of the polymer is also closely linked to its chain conformation in solution, the solubility parameter can be determined by the fluorescence intensity. The range of the solubility parameter of polymethyl methacrylate (PMMA) tested by this method was from 9.00 cal^1/2^cm^−3/2^ to 10.00 cal^1/2^cm^−3/2^. The results are more accurate than those obtained from the traditional turbidimetric titration method, ranging from 8.60 cal^1/2^cm^−3/2^ to 12.15 cal^1/2^cm^−3/2^. According to the photoluminescence (PL) intensities spectra, the solubility parameters of PMMA and polyvinyl acetate (PVAc) are 9.19 cal^1/2^cm^−3/2^ and 9.85 cal^1/2^cm^−3/2^, respectively.

## 1. Introduction

Luminescent materials are rapidly being developed and have aroused great academic interest [1,2,3]. Generally, a luminescent substance in the state of aggregation would produce two different kinds of photoluminescence (PL) effects: aggregation-induced emission (AIE) and aggregation-caused quenching (ACQ) [4,5,6]. When the AIE molecules aggregate in concentrated solutions or on surfaces of solid materials, they become highly luminescent. Thus, the AIE phenomenon can be applied in some special fields [7,8].

The solubility parameter is defined as the square root of the total energy of vaporization of the liquid [9]. Since polymers would decompose before the heat of the vaporization could be measured, the solubility parameter of polymer is measured indirectly, and it can be estimated from experimental testing methods such as turbidimetric titration [10,11], viscometry [12], the swelling method [13], etc. The interactions of polymer chains and solvent molecules are most conveniently described by the Flory-Huggins (F-H) model [14,15], and the interaction parameters χ and the second virial coefficient A_2_ are closely related to the conformations of polymer chains in solution [16]. When a polymer dissolves in a good solvent, the polymer chains are in stretched conformations, A_2_ > 0, χ < 1/2; with a bad solvent added, the polymer chains are in entangled conformations, A_2_ < 0, χ > 1/2. On the basis of the Hildebrand approximation [17], polymer chains are in stretched conformations when the solubility parameters of the solvent and polymer are similar. When choosing a good solvent for the polymer, a mixed solvent is often adopted rather than a single solvent. The solubility parameter of the mixed solvent δ_mix_ can be adjusted to an accurate value by Equation (1), as follows:

δ_mix_ = δ_1_φ_1_ + δ_2_φ_2_ (1)

where δ_1_, δ_2_ are the solubility parameters of two types of pure solvents; φ_1_, φ_2_ are the volume fractions of two types of pure solvents. In addition to considering whether the solubility parameters of the solvent and polymer are similar, some physical parameters such as the dispersion force, polarity force, and hydrogen bond should be taken into account when choosing a good solvent for polymers.

**Scheme 1:**
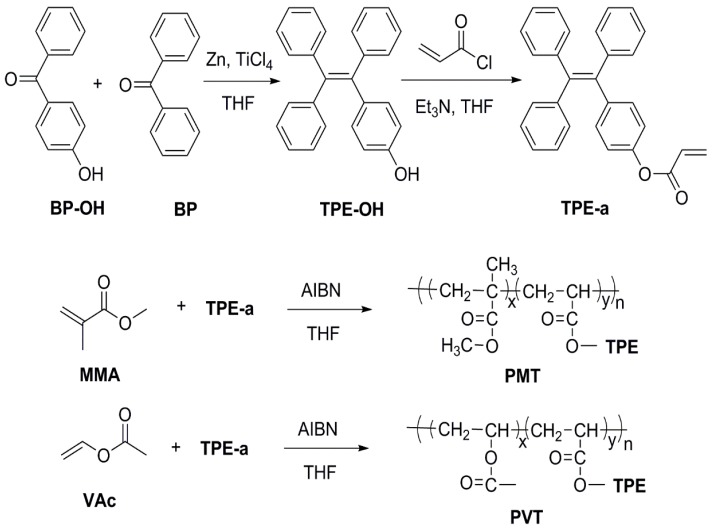
Synthesis of aggregation-induced emission (AIE) polymers PMT and PVT.

In our work, a luminescence probe based on 4-Acryloyl tetraphenylethylene (TPE-a) [18] was introduced into the polymers polymethyl methacrylate(PMMA) and polyvinyl acetate(PVAc) by radical copolymerizations, giving birth to AIE polymers P(MMA-co-TPE-a) (PMT) and P(VAc-co-TPE-a) (PVT) (Scheme 1). The solubility parameter of the solvent, in which the polymer chains are in fully stretched conformations, can serve as the solubility parameter of the polymer itself. The state of the polymer chains was observed continuously by luminescent intensity in a series of solvents with different solubility parameters.

## 2. Results and Discussion

**Figure 1:**
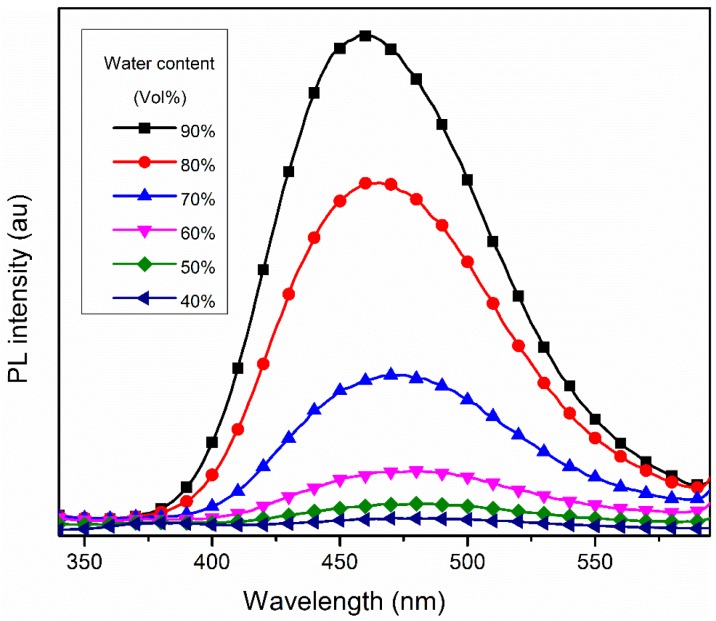
Photoluminescence (PL) spectra of PMT in THF-water mixtures with different water contents (λ_ex_ = 310 nm, [P] = 1 mg·mL^−1^).

The PL spectra of PMT in THF-water mixtures with different water contents are shown in Figure 1. When a large amount of water (50% vol.) was added to the THF solution, an emission peak emerged at 465 nm, demonstrating a typical AIE phenomenon. Despite being weak, the polymer is somewhat fluorescent in the THF solution. The emission became stronger progressively with increasing the amount of water in the mixed solvent. During the process, the solubility of the PMT got worse and the polymer chains changed from stretched conformations to curled conformations. At 90% vol. water content, the PL intensity is clearly higher than that of the 50% vol. water content solution, while the emission maximum is still located at 465 nm.

**Figure 2:**
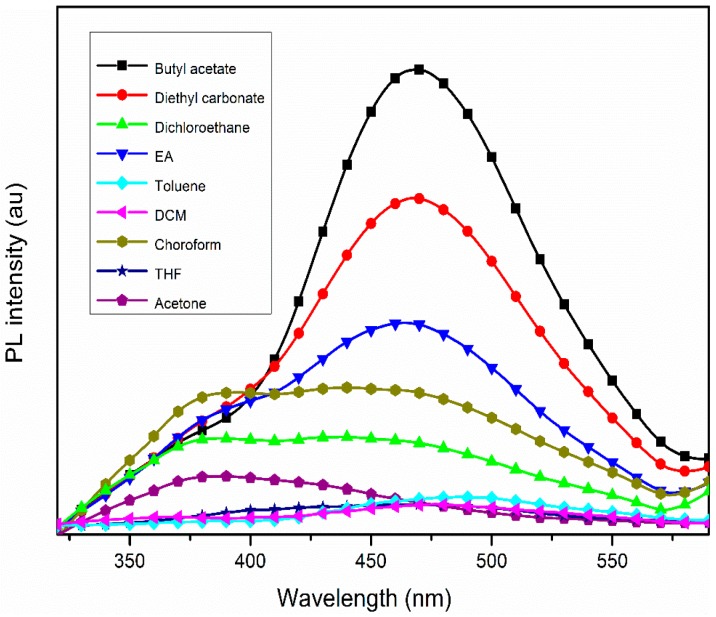
PL spectra of PMT in different solvents (λ_ex_ = 310 nm, [P] = 1 mg·mL^−1^).

Different kinds of solvents were chosen to dissolve PMT. Although all these solutions were transparent, the PL spectra presented big differences. From the PL spectra (Figure 2), when PMT was dissolved in dichloromethane (DCM), the PL intensity reached the minimum at 465 nm. Therefore, DCM is considered as a good solvent for PMT.

**Figure 3:**
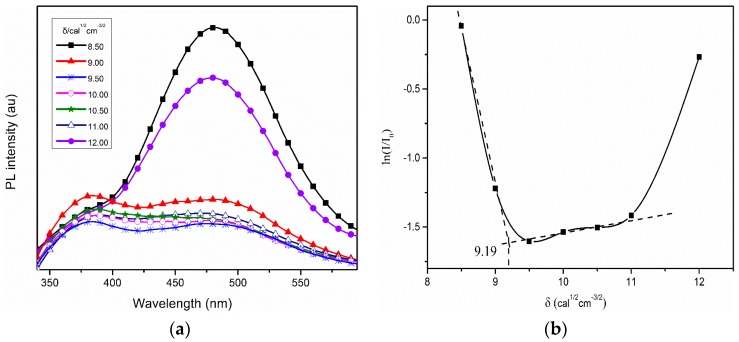
(**a**) PL spectra of PMT in mixed solvents with different solubility parameters (δ = 8.50~12.00 cal^1/2^cm^−3/2^, λ_ex_ = 310 nm, [P] = 1 mg·mL^−1^); (**b**) Plots of ln(I/I_0_) of PL spectra (**a**).

Then, DCM was mixed with cyclohexane and ethyl alcohol in varying proportions, giving birth to a series of solvents with different solubility parameters (Figure 3a). It was found that when the δ of mixed solvent was below 9.00 cal^1/2^cm^−3/2^ or above 10.00 cal^1/2^cm^−3/2^, the PL intensity and the relative fluorescent quantum yield (ln(I/I_0_)) (Figure 3b) changed monotonically, indicating that the polymer chains tend to shrink. Additionally, when the δ was below 8.50 cal^1/2^cm^−3/2^ or above 12.00 cal^1/2^cm^−3/2^, the polymer precipitated thoroughly. From the range of 9.00 cal^1/2^cm^−3/2^ to 10.00 cal^1/2^cm^−3/2^, the PL intensity and ln(I/I_0_) decreased at first, and then they increased, indicating the minimum intensity must be in this range. Based on the analysis, the intersection point of the trendline on both sides of 9.50 cal^1/2^cm^−3/2^ refers to δ = 9.19 cal^1/2^cm^−3/2^ (Figure 3b), which can be considered as the solubility parameter of PMT. Furthermore, since the molar content of TPE units is only 0.47%, which is extremely low, its impact on the solubility parameter could be neglected and the solubility parameters of PMT and PMMA are the same.

For comparing the fluorescence probe technique with the turbidimetric titration method, which is now the most commonly used method for determining the solubility parameter of a polymer, the turbidimetric titration was carried out by measuring the luminousness of the solutions to estimate the cloud point. The solubility parameter of PMT obtained by the turbidimetric titration method ranged from 8.60 cal^1/2^cm^−3/2^ to 12.15 cal^1/2^cm^−3/2^, which is much wider than the range (9.00 cal^1/2^cm^−3/2^ to 10.00 cal^1/2^cm^−3/2^) determined by our fluorescence method above.

**Figure 4:**
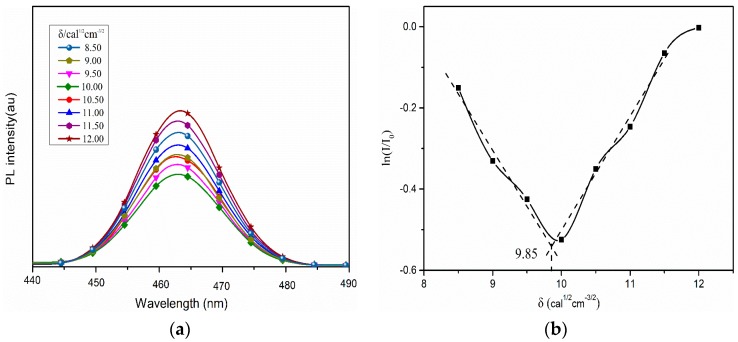
(**a**) PL spectra of PVT in mixed solvents with different solubility parameters (δ = 8.50~12.00 cal^1/2^cm^−3/2^, λ_ex_ = 310 nm, [P] = 1 mg·mL^−1^); (**b**) Plots of ln(I/I_0_) of PL spectra (**a**).

To further explore the application of the fluorescence method, the solubility parameter of PVT was also determined. The n-hexane, DCM and ethyl alcohol were used for preparing mixed solvents with different solubility parameters to measure the solubility parameter of PVT (Figure 4a). When the δ of the mixed solvent was 10.00 cal^1/2^cm^−3/2^, the PL intensity of solution reached the relative minimum at 463 nm. The intensity and ln(I/I_0_) changed monotonically below 9.50 cal^1/2^cm^−3/2^ or above 10.50 cal^1/2^cm^−3/2^. From the range of 9.50 cal^1/2^cm^−3/2^ to 10.50 cal^1/2^cm^−3/2^, the PL intensity and ln(I/I_0_) decreased at first, and then they increased, indicating the minimum intensity must be in this range. Figure 4b shows that the intersection point of the trendline on both sides of 10.00 cal^1/2^cm^−3/2^ refers to δ = 9.85 cal^1/2^cm^−3/2^, which can be considered as the solubility parameter of PVT. Since the molar content of TPE units is only 0.37%, which is extremely low, the solubility parameter of PVT could be identical to the solubility parameter of PVAc.

## 3. Materials and Methods

### 3.1. General

Tetrahydrofuran (THF), methyl methacrylate (MMA), vinyl acetate (VAc), and triethylamine (Et_3_N) were freshly purified, respectively. Azobisisobutyronitrile (AIBN) was purified by recrystallization. 4-hydroxylbenzophenone, benzophenone, and acryloyl chloride were purchased from Aladdin Industrial Co., (Fengxian, Shanghai, China) and used as received without further purification. Number-average molecular weight and polydispersity of polymers by GPC (Waters Co., Milford, MA, USA) and THF as the eluent. NMR spectra were measured on AVANCE III 400 MHz (Bruker Co., Fällanden, Switzerland). PL spectra were measured on a PerkinElmer LS45 spectrometer (Fremont, CA, USA).

### 3.2. Synthesis of 4-Hydroxyl Tetraphenylethylene

Into a 500 mL two-necked round-bottom flask were added 4-hydroxylbenzophenone (1.9 g, 10 mmol), benzophenone (2.2 g, 12 mmol) and Zn powder (2.9 g, 44 mmol). The flask was evacuated under vacuum and flushed with dry N_2_ three times. Then 80 mL fresh dried THF was added. After all the solids were completely dissolved, the solution was cooled to 0 °C and TiCl_4_ (2.4 mL, 22 mmol) was slowly injected into the flask. After refluxing at 70 °C overnight, the mixture was cooled to room temperature and 80 mL dilute hydrochloric acid (1 mol·L^−1^) was added to it, which was extracted with dichloromethane (3 × 80 mL). The collected organic layer was dried over anhydrous magnesium sulfate. After solvent evaporated, the crude product was purified by a silica gel column chromatography using petroleum ether (60–90 °C)/ethyl acetate (40:1) as eluent. White crystal; yield 58% (2.4 g). ^1^H-NMR (400 MHz, CDCl_3_, δ): 7.16–7.09 (m, 8H), 7.07–7.01 (m, 9H), 6.91 (d, 1H), 6.58 (d, 1H), 4.78 (s, 1H).

### 3.3. Synthesis of 4-Acryloyl Tetraphenylethylene

Into a 250 mL two-necked round-bottom flask were added 4-Hydroxyl Tetraphenylethylene (TPE-OH) (1.0 g, 3 mmol), trimethylamine (2 mL) and fresh dried THF (30 mL). After all the solids were completely dissolved, acryloyl chloride (0.3 mL, 3.6 mmol) was injected into the flask dropwise over 20 min. After stirring at 0 °C for 2 h, the reaction mixture was concentrated under vacuum. The residue was dissolved in ethyl acetate and the resulting solution was washed with water and brine, dried with Na_2_SO_4_ and concentrated. The crude product was purified by a silica gel column chromatography using petroleum ether (60–90 °C)/ethyl acetate (100:1) as eluent. White crystal; yield 62% (0.83 g). ^1^H-NMR (400 MHz, CDCl_3_, δ):7.14–7.07 (m, 9H), 7.05–6.99 (m, 8H), 6.88 (d, 1H), 6.55 (d, 1H), 6.31–6.24 (q, 1H), 5.99 (d, 1H), 5.97 (d, 1H).

### 3.4. Synthesis of PMT and PVT

The polymerization reaction was carried out by radical polymerization under N_2_ and equipped with a condenser and magnetic stirring. Into 500 mL three-necked round-bottom flask were added TPE-a (0.3 g, 0.7 mmol), MMA (15 g, 150 mmol), AIBN (0.1 g, 0.6 mmol) and DMF (80 mL). The reaction mixture was stirred on an oil bath at 70 °C under nitrogen for 16 h. The solution was then cooled to room temperature, concentrated under vacuum and added dropwise to 500 mL of methanol under stirring to precipitate the polymer and meanwhile remove the unreacted reactants and solvent. The polymer was filtered by Buchner funnel, washed with methanol several times, and dried in vacuum overnight at 40 °C to a constant weight. White solid; yield 86% (13.2 g). M_n_: 16500, PDI: 1.78 (GPC); ^1^H-NMR (400 MHz, CDCl_3_, δ): 7.13–6.99 (multi-peak of TPE), molar ratio of TPE: 0.47%.

The PVT was synthesized using the same synthesis methods of PMT. White solid; yield 82% (12.6 g). M_n_: 12800, PDI: 1.68 (GPC); ^1^H-NMR (400 MHz, CDCl_3_, δ): 7.13–6.98 (multi-peak of TPE), molar ratio of TPE: 0.37%.

## 4. Conclusions

We have demonstrated a luminescence probe approach based on the AIE molecule to measure the solubility parameters of PMMA and PVAc. The solubility parameters of PMMA and PVAc are 9.19 cal^1/2^cm^−3/2^ and 9.85 cal^1/2^cm^−3/2^, respectively. The measured parameter of PMMA ranges from 9.00 cal^1/2^cm^−3/2^ to 10.00 cal^1/2^cm^−3/2^, while the solubility parameter of PMMA measured by the turbidimetric titration ranges from 8.6 cal^1/2^cm^−3/2^ to 12.15 cal^1/2^cm^−3/2^. All these results demonstrated that the values obtained from our method should be more accurate than those obtained by the turbidimetric titration method, and the luminescence probe approach can also be applied to other kinds of polymers.
